# c-Jun N-terminal Kinase (JNK) Signaling as a Therapeutic Target for Alzheimer’s Disease

**DOI:** 10.3389/fphar.2015.00321

**Published:** 2016-01-12

**Authors:** Ramon Yarza, Silvia Vela, Maite Solas, Maria J. Ramirez

**Affiliations:** ^1^Department of Pharmacology and Toxicology, University of NavarraPamplona, Spain; ^2^Navarra Institute for Health ResearchPamplona, Spain

**Keywords:** apoptosis, βamyloid, tau, kinase, inhibitors, SP600125, D-JNKI1

## Abstract

c-Jun N-terminal kinases (JNKs) are a family of protein kinases that play a central role in stress signaling pathways implicated in gene expression, neuronal plasticity, regeneration, cell death, and regulation of cellular senescence. It has been shown that there is a JNK pathway activation after exposure to different stressing factors, including cytokines, growth factors, oxidative stress, unfolded protein response signals or Aβ peptides. Altogether, JNKs have become a focus of screening strategies searching for new therapeutic approaches to diabetes, cancer or liver diseases. In addition, activation of JNK has been identified as a key element responsible for the regulation of apoptosis signals and therefore, it is critical for pathological cell death associated with neurodegenerative diseases and, among them, with Alzheimer’s disease (AD). In addition, *in vitro* and *in vivo* studies have reported alterations of JNK pathways potentially associated with pathogenesis and neuronal death in AD. JNK’s, particularly JNK3, not only enhance Aβ production, moreover it plays a key role in the maturation and development of neurofibrillary tangles. This review aims to explain the rationale behind testing therapies based on inhibition of JNK signaling for AD in terms of current knowledge about the pathophysiology of the disease. Keeping in mind that JNK3 is specifically expressed in the brain and activated by stress-stimuli, it is possible to hypothesize that inhibition of JNK3 might be considered as a potential target for treating neurodegenerative mechanisms associated with AD.

## Introduction

Since its discovery more than 20 years ago, the c-Jun N-terminal kinase family (JNK) has remained a subject of intense research interest with continued efforts to evaluate its biochemistry and regulation, and its contribution to cellular events under physiological and pathophysiological conditions. The JNK family of protein kinases is one of the three identified families of mitogen activated protein (MAP) kinases. Three genes, namely *jnk1* (MAPK8), *jnk2* (MAPK9), and *jnk3* (MAPK10), encode for 10 different splice variants with molecular weights of 46 and 55 kDa (Davis, 2000). Whereas, JNK1 and JNK2 have a broad tissue distribution, JNK3 is mainly localized in neurons and to a lesser extent in the heart and the testis.

The discovery of JNK pathway scaffolds such as JNK-interacting protein-1 (JIP1) and related proteins, as well as the identification of JNK inhibitors have contributed to unmask the roles for the JNKs in both normal physiology and disease. JNK signaling process has been studied as an active pathological mechanism in many different diseases, especially in the field of oncology. To mention a few, JNK has been involved in regulation of the natural killer cells’ cytokine production and secretion (Lee et al., 2014), oncology models and drug-resistant tumor cells (Chuang et al., 2014; Kim et al., 2014; Okada et al., 2014; Volk et al., 2014) or myeloproliferative disorders (Funakoshi-Tago et al., 2012).

Transgenic knockouts of JNK isoforms have provided crucial insights into the roles played in the brain by each JNK isoform. It has been established that JNK1 and JNK2 have important roles in the modulation of immune cell function and in the development of the embryonic nervous system. A study using JNK1 knockout mice demonstrated that JNK1 has a regulatory role and maintains physiological functions in the CNS, while JNK2 knockout established that this isoform may also participate in some physiological functions and, particularly, in the long term potentiation (LTP; Chen et al., 2005). JNK3 is a multifunctional enzyme important in controlling brain functions under both normal and pathological conditions. JNK3 has been implicated in brain development (Kuan et al., 1999), neurite formation and plasticity (Waetzig et al., 2006; Eminel et al., 2008), in addition to memory and learning (Bevilaqua et al., 2003; Brecht et al., 2005). Under pathological conditions, JNK3 has been considered as a degenerative signal transducer and it seems to be the isoform involved in over-activation of JNK after deleterious stress-stimuli in adult brain (ischemia, hypoxia, epilepsies). This principle is supported by the data on the reduced apoptosis of hippocampal neurons and reduced seizures induced by kainic acid in JNK3 knockout (–/–) mice, and by the notion that JNK3–/– mice are also protected against ischemia (Yang et al., 1997; Okazawa and Estus, 2002; Sahara et al., 2008). Therefore, there is now considerable interest in further studying the involvement of this isoform in the development of neurodegenerative disease, such as Alzheimer’s disease (AD).

## JNK Signaling

**FIGURE 1:**
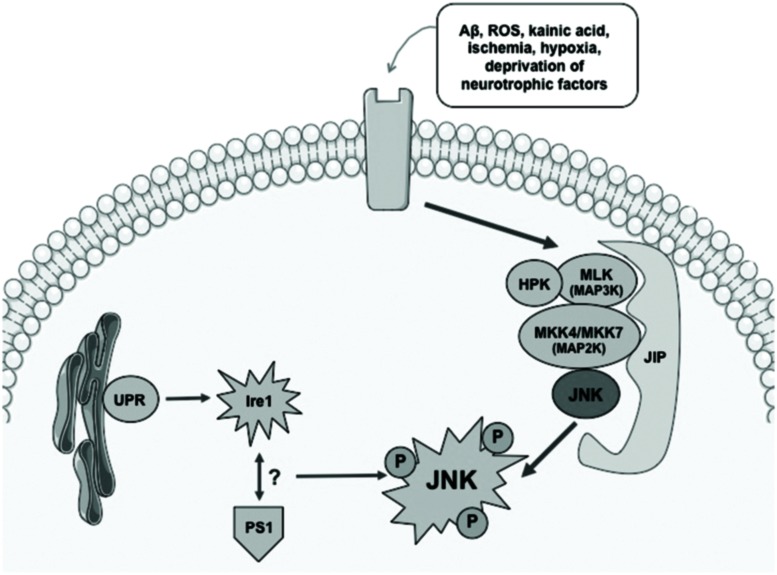
**Simplified diagram showing mechanisms involved in activation of the JNK pathway**. Different stress conditions might activate JNK signaling via scaffold proteins. UPR and an interaction between Ire1 and PS1 have also been described as potential activators of JNK. ROS, radical oxygen species; Aβ, βamyloid; JIP, JNK interacting protein; UPR, unfolded protein response; Ire, endoplasmic reticulum to nucleus signaling 1.

Activation of the JNK pathway relies on the coordinated interaction of the scaffold proteins belonging to the JNK activation complex. These proteins are able to mediate the biochemical signal amplification and also to ensure substrate-specificity as well as a coordinated cascade signaling (**Figure 1**). The interaction between scaffold proteins leads to the activation of JNK by bi-phosphorylating different substrates, enables the activation of different functions (Antoniou et al., 2011).

Different stimuli that have been described as able to trigger the signaling response to JNK include nerve growth factor (NGF) deprivation, trophic support withdrawal, DNA damage, oxidative stress, βamyloid (Aβ) exposure, low potassium, excitotoxic stress, 6-OHDA, UV irradiation, tumor necrosis factor (TNF), or the Wnt cascade Mudher et al., 2001; Cui et al., 2007). Many are the scaffold proteins that have been described as the signaling proteins that converge in the activation of JNK: JIP1a (JNK interacting protein 1a) and JIP1b (also named IB1), JIP2 and JIP3 (firstly named JSAP1) JNK-interacting leucine zipper protein (JLP) and plenty of SH3 (POSH; Engstrom et al., 2010). JIPs belong to second-order-activating proteins that are dependent on previous interaction with MAPK activating kinases (MAPKKs) and MAPKK activating kinases (MAPKKKs; Wang et al., 2004; Cui et al., 2007; Engstrom et al., 2010; **Figure 1**). Thus, the coordination of what is called the “signalosome” that leads to the activation of JNK is complex and requires interaction of first messengers at different cellular levels for further activation of the scaffold-protein-complex and finally activating JNK.

Endoplasmic reticulum’s (ER) stress phenomena that induce the unfolded protein response (UPR) signaling are also involved in the control of activation of JNK pathway (**Figure 1**). As a result of anomalous protein burden, an interaction between Ire1 (ER to nucleus signaling 1) and Presenilin 1 (PS1) has been proposed to enable the activation of JNK thus leading to proapoptotic signaling activation (Shoji et al., 2000). Direct modulation of JNK-activation by the cdk5/p35 complex has also been described, although the underlying mechanisms that lead to this molecular phenomenon are still unclear (Otth et al., 2003).

**FIGURE 2:**
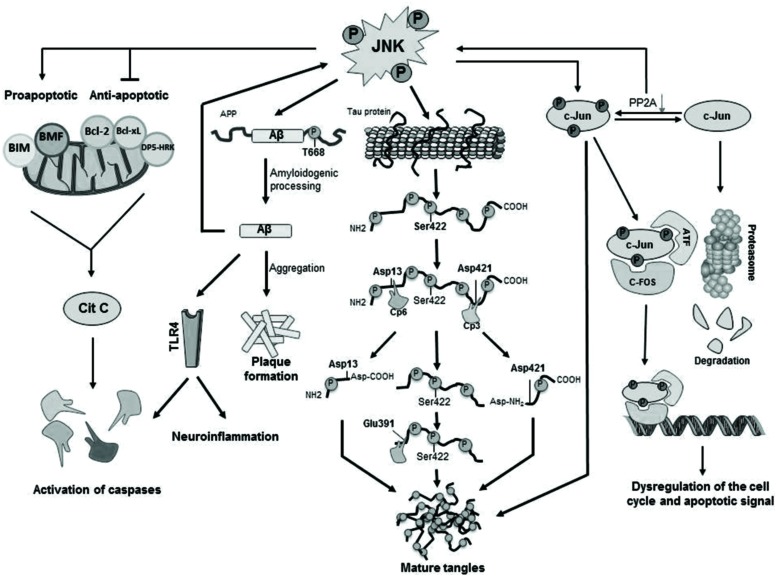
**Cellular mechanisms activated by JNK phosphorylation**. Activated JNK leads to phosphorylation of c-Jun, which modulates gene expression as well as tangle maturation. JNK plays also a direct role in the formation of tangles by phosphorylation of Tau and it also contributes to the regulation of PHF formation and proteolytic cleavage processing. Finally, JNK is responsible for the phosphorylation of BIM and BMF and, as consequence, for the activation of caspases leading to cellular apoptosis.

The main cellular substrate activated by JNK mediated phosphorylation is c-Jun (**Figure 2**), which in turn is able to interact with JunB, JunD, c-Fos, and ATF constituting the AP-1 transcription factor (Pearson et al., 2006; Cui et al., 2007) and, thus, regulating maturation of the cellular stress-response or modulating the signals that lead finally to activation of caspases (Nishina et al., 2004; Pearson et al., 2006). Moreover, JNK is able to phosphorylate and activate directly apoptosis-related proteins such as BIM (homologous to BAX) and BMF (Okazawa and Estus, 2002; Cui et al., 2007), both proapoptotic proteins resulting in activation of caspases. JNK also phosphorylates DP5-HRK, Bcl-2, and Bcl-xL (Okazawa and Estus, 2002; Cui et al., 2007), which are anti-apoptotic proteins inhibited by phosphorylation by JNK (**Figure 2**).

Furthermore, it has also been described that JNK might exert its effects via microRNA (miRNA) mechanisms or regulation of histone H3 acetylation, as reviewed by Bogoyevitch et al. (2010).

## JNK and Alzheimer’S Disease

Alzheimer disease is an age-related neurodegenerative disorder clinically characterized by progressive deterioration of cognitive functions. At cellular level, major neuropathological lesions of AD include extracellular deposits of Aβ peptides leading to formation of senile/neuritic plaques and intracellular neurofibrillary tangles (NFTs) which are paired helical filaments (PHFs) of hyper-phosphorylated tau proteins (Haas, 2012).

It has been shown an increased expression of phosphorylated JNK (pJNK) in human post-mortem brain samples from AD patients and a positive co-localization with Aβ (Zhu et al., 2001; Killick et al., 2014). In particular, JNK3 is highly expressed and activated in brain tissue and cerebrospinal fluid from patients with AD and statistically correlated with the rate of cognitive decline (Gourmaud et al., 2015). In fact, it has been described that Aβ peptides are able to induce JNK activation, as it has been found *in vitro* that p JNK increases after treatment with Aβ in primary cortical and hippocampal cultures from C57BL/6 mice, in primary cortical cell cultures from Wistar rat and in SH-SY5Y neuroblastoma cells (Morishima et al., 2001; Suwanna et al., 2014; Xu et al., 2015). Interestingly, Yoon et al. (2012) demonstrated that JNK3 is the major kinase for β-amyloid precursor protein (APP) phosphorylation at T668 (**Figure 2**). In fact, genetic depletion of JNK3 in transgenic AD mice resulted in a dramatic reduction in Aβ42 peptide levels and overall plaque loads as well as in an increased number of neurons and improved cognition (Yoon et al., 2012). Some reports confirmed that JNK3-mediated phosphorylation regulated APP cleavage by inducing the amyloidogenic processing of the protein, while JNK inhibition reduced amyloidogenic processing in favor of the non-amyloidogenic route *in vitro* by blocking APP phosphorylation (Morishima et al., 2001; Savage et al., 2002; Colombo et al., 2009).

In experimental models of AD, research using a mouse model of AD that incorporates the Swedish APP mutation and a mutant presenilin-1 (PS1) -Tg2576/PS1- has demonstrated that JNK activation is associated with increased levels of senile plaques and NFT’s (Savage et al., 2002). However, in contrast with these data, no significant differences were found in pJNK levels in the triple transgenic mice (3xTg mice; Feld et al., 2014). Research has also been conducted in experimental models of AD based on well-known risk factors contributing to the development of AD, such as stress or insulin resistance (Dhikav and Anand, 2007). In mice subjected to chronic mild stress (CMS) known to increase tau misprocessing and amyloidogenic processing, JNK phosphorylation is increased (Solas et al., 2013a). The intracerebroventricular administration of subdiabetogenic doses of streptozotocin (STZ) induced cognitive and brain cholinergic deficits, oxidative stress, insulin resistant brain state and high levels of pJNK (Giuliani et al., 2013; Martisova et al., 2013; Salkovic-Petrisic et al., 2013; Solas et al., 2013b; Xiong et al., 2013). It is to be noted that JNK may also directly induce insulin resistance, as JNK phosphorylates insulin receptor substrate (IRS) 1 blocking the transduction signal produced by the insulin receptor (Sabio et al., 2008).

The etiology of AD remains elusive, but the nosogenic basis of AD seems to be related to neuron apoptosis and loss of synaptic terminals within the central nervous system’s parenchyma. Thus, the increased concentration of reactive oxygen intermediates (ROIs) and superoxide dismutase, both markers of cellular stress, and increased intracellular calcium in AD are congruent with an underlying activation of apoptotic mechanisms via mitochondrial dysfunction. However, the molecular mechanisms that lead to the activation of apoptotic signals are not fully understood. There is evidence that Aβ42 induces a translational block leading to activation of JNK (Yoon et al., 2012), which in turn, results in neuroinflammation and neurodegeneration. It has been suggested that neurodegeneration in early age of AD patients could be a result of an increased vulnerability of neurons through activation of different apoptotic pathways as a consequence of elevated levels of oxidative stress, and that these effects could be mediated by JNK activation (Marques et al., 2003; Sahara et al., 2008). Furthermore, JNKs were involved in Aβ triggered down regulation of the anti-apoptotic Bcl-w (Yao et al., 2005) and activation of Toll-like receptor 4 (TLR4) signaling (**Figure 2**). Neurons from TLR4 mutant mice exhibit reduced JNK and caspase-3 activation and protect against Aβ induced apoptosis (Tang et al., 2008).

c-Jun has been identified to play other possible roles in AD, e.g., phosphorylated c-Jun burdens within the structure of NFTs may play an indirect regulatory role in tangle maturation in AD, mostly regulated by its phosphorylation by JNK. Due to the imbalance established between decreased PP2A (protein phosphatase 2) expression and JNK mediated phosphorylation of c-Jun, phospho-c-Jun are preponderant over the non-phosphorylated form. As a matter of fact, phospho-c-Jun shows a lesser tendency for its degradation via proteasomes, leading to its accumulation within NFTs and, thus, contributing to tangle maturation process (Pearson et al., 2006; **Figure 2**).

c-Jun N-terminal kinase also modulates directly the formation of NFTs (**Figure 2**) by direct phosphorylation of Tau (Lagalwar et al., 2006). *In vitro* phosphorylation experiments show that the JNK3 isoform can strongly autophosphorylate itself and contribute to Tau hyperphosphorylation (Vogel et al., 2009). JNK was identified to phosphorylate Tau at Ser^422^, and concretely, JNK3 has the highest affinity toward phosphorylation at Ser^422^ (Yoshida et al., 2004) thus regulating hydrolysis at Asp^421^ by caspase-3. In fact, phosphorylation at Ser^422^ has shown to protect against caspase hydrolysis at Asp^421^ (Guillozet-Bongaarts et al., 2005, 2006; Kolarova et al., 2012). In physiological conditions Tau is responsible not only for the stabilization of neuronal cytoskeleton by its binding to tubulin monomers but also for many intra and extracellular signaling processes (Kolarova et al., 2012).

## Novel Compounds Targeting JNK Inhibition

Inhibition of JNKs is an attractive therapeutic strategy that has been investigated with considerable recent effort from both the pharmaceutical industry and academia. The development of JNK inhibitors prior to 2010 has been extensively reviewed by Siddiqui and Reddy (2010). A recent review of patents claiming inhibitors of all JNK isoforms published between 2010 and 2014 can be consulted (Gehringer et al., 2015).

**FIGURE 3:**
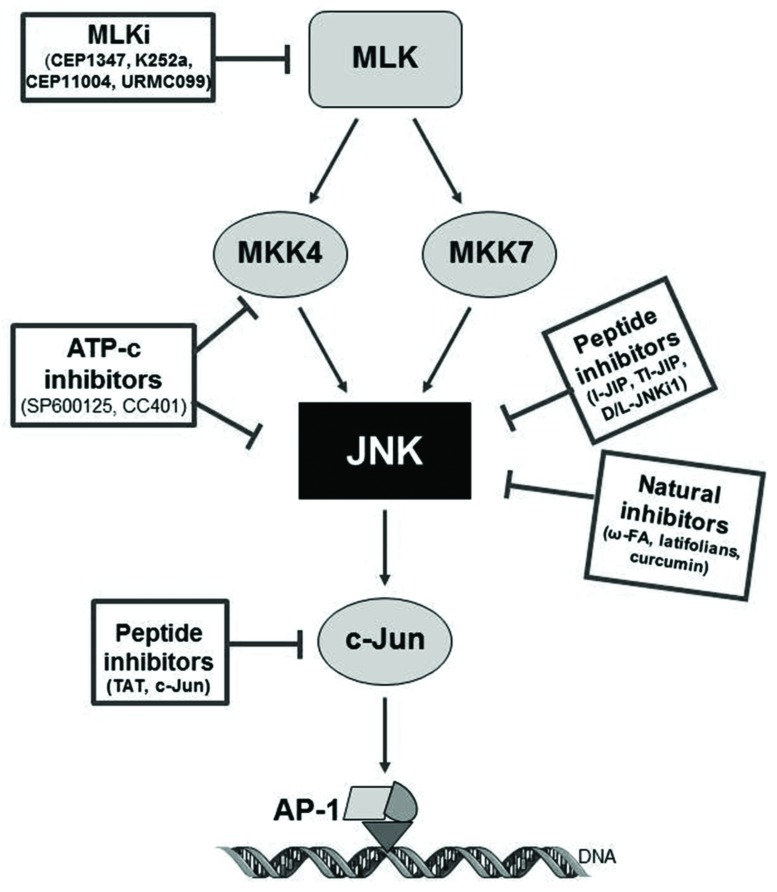
**Pharmacology of JNK inhibitors, targets and mechanism of action**. CPPi, cell-permeable peptide inhibitors; ATP-c, direct ATP-competitive inhibitors; MLKi, mixed linage kinase inhibitors.

Within the past years, few small molecule inhibitors of JNKs have entered clinical trials for different indications, but none for the treatment of AD. Current compounds under evaluation are: bentamapimod for the treatment of inflammatory endometriosis, CC-930 (tanzisertib) for the treatment of idiopathic pulmonary fibrosis and discoid lupus erythematous as well as D-JNKi1 for the treatment of inflammation and stroke (as reviewed by Koch et al., 2015). In the following sections, and represented in **Tables 1** and **2** and **Figure 3**, current knowledge of the JNK inhibitors will be described (Bogoyevitch et al., 2004; Wang et al., 2004; Bogoyevitch and Arthur, 2008; Antoniou et al., 2011).

**Table 1:** Use of SP600125 as a possible therapeutic target in Alzheimer’s disease.

	Experimental model	Findings	Reference
*In vitro*	F11 cells	Blockade of βAPP dimerization and ASK1 (MAP3K5) mediated neuronal cell death	Hashimoto et al., 2003
	Hippocampal cell culture from Wistar rats	Increased synaptic transmission in CA1 region	Costello and Herron, 2004
	Murine L929 fibroblasts	Block of τ phosphorylation induced by WOX1 knock-down in cell culture	Sze et al., 2004
	Primary rat microglia culture	Reduced nitrite accumulation and prevention of iNOS’s activation in glial cells	Bodles and Barger, 2005
	Primary cortical cell culture from Sprague Dawley rat	Inhibition of Bcl-w and Bcl-xL down-regulation	Yao et al., 2005
	Neuroglioma U251 cells	Inhibition of IL1β induced sAPPα release	Ma et al., 2005
	PC12 cells	Attenuation of 4-hydroxynonenal induced apoptosis	Cho et al., 2009
	Cultured human brain endothelial cells	Inhibition of Aβ induced AP-1 activation and MCP1	Vukic et al., 2009
	Primary rat hippocampal culture	Inhibition of both hetero- and autophosphorylation of JNK	Vogel et al., 2009
	Human neuroglioma H4 cells expressing Swedish APP695 or intracellular APP C99	Inhibition of staurosporine-induced Aβ	Chae et al., 2010
	SK-N-SH cell line	Reduction of passive calcium leak in endoplasmic reticulum	Das et al., 2012
	Primary cortical cell culture from Sprague Dawley rat	Reduction of morphine induced τ phosphorylation	Cao et al., 2013
	CMEC/D3 cells	Reduction of Aβ induced cytokine expression	Bamji-Mirza et al., 2014
	Primary glial culture from Swiss-Webster mice	Increase of ApoE/ABCA1 expression	Pocivavsek and Rebeck, 2009
*In vivo*	Male Swiss-Webster mice	Increase of ApoE/ABCA1 expression	Pocivavsek and Rebeck, 2009
	Male albino Wistar rats	Improvement of escape latency on Morris Water Maze	Ramin et al., 2011
	*Drosophila* sp. fly strains	Rescue of Aβ42 induced apoptosis	Hong et al., 2012
	Male C57BL/6 mice	Reduction of PS1 expression	Rahman et al., 2012
	Sprague Dawley neonatal rats	Attenuate isoflurane-induced hippocampal apoptosis mediated by JNK	Li et al., 2013
	APPswe/PS1dE9 mice	Reversion of synaptic loss, decrease of IL1β, IL6 and TNFα expression, decrease of phosphorylated τ, increase of αAPP, decrease of βAPP and Aβ oligomers and improvement of spatial learning	Zhou et al., 2015

**Table 2:** Use of different JNK inhibitors as a possible therapeutic target in Alzheimer’s disease.

JNK Inhibitor	Experimental Model	Findings	Reference
***Mixed linage kinase inhibitors***			
K252a	Primary cell culture	Conferred neuroprotection to Aβ-exposition	Goodman and Mattson, 1994
	Primary cell culture	Prevention against Aβ-induced neuroapoptosis	Xu et al., 2009
CEP1347	Primary cell culture	Prevention against Aβ-induced neuronal cell	Bozyczko-Coyne et al., 2001
	PC12 cell cultures	Prevention against Aβ-induced neuronal cell death	Troy et al., 2001
***Peptide inhibitors***			
TAT-TIJIP	Primary cell culture	Prevention against neuronal apoptosis	Meade et al., 2010
	Primary cell culture	Decrease of neuronal degeneration and dendrite loss	Meloni et al., 2014
D-JNKi1	TgCRND8 mice	Decrease of APP phosphorylation. Improvement of memory	Sclip et al., 2011
	3xTg-AD mice with traumatic brain injury	Prevention of Tau phosphorylation	Tran et al., 2012
	TgCRND8 mice	Decrease of synaptic loss and preventing synaptic dysfunction	Sclip et al., 2014
	C57BL/6J mice + corticosterone regimen	Decrease of pTau levels and neuronal cell death	Solas et al., 2013b
***Natural inhibitors***			
ω-fatty acids	Tg2576 mice on DHA regimen	Decreased PI3K activity. Increase of caspase-cleaved actin	Calon et al., 2004
	C57BL/6J mice on DHA regimen	Decrease of both γ and β-secretase activity	Grimm et al., 2011
	Tg2576 mice on DHA regimen	Decrease of Aβ levels	Lim et al., 2005
	Neuronal cell culture	Prevented IRS-1 inactivation and pTau pathology	Ma et al., 2009
Curcumin	3xTg-AD mice	Reduced Aβ, plaque deposition, and cytoquines levels	Ma et al., 2009
	APPswe/PS1dE9 mice	Reduced hippocampal Aβ40/42 levels	Feng et al., 2014
	APPswe/PS1dE9 mice	Spatial learning and memory improvements. Reduced hippocampal Aβ levels	Wang et al., 2014
	Tg2576 mice	Reduced amyloid levels and plaque burden. Direct Aβ-binding prevention of fibril formation and aggregation	Yang et al., 2005

*DHA, Docosahexaenoic acid; PI3K, phosphoinositol 3 kinase*.

### Direct ATP-Competitive Inhibitors: SP600125

Since the first JNK inhibitors were synthesized, the use of SP600125 (Anthra[1,9-cd]pyrazol-6-(2H)-one; Bogoyevitch et al., 2004) has been increasingly used in order to understand and elucidate the role of JNK in pathological conditions and a purported therapeutic role of SP600125 (i.e., Zhou et al., 2015). However, it is to note that SP600125 has shown a limited specificity toward JNK, as also inhibits not only MKK4 and MKK7, but also other protein kinases unrelated to JNK, such as SGK, p70 ribosomal protein S6 kinase (S6K1), AMPK, CDK2, CK1d, and DYRK1A (Bain et al., 2003).

In both *in vitro* and *in vivo* models of AD, SP600125 has demonstrated to prevent the pathological mechanisms triggered by the up-regulation of pJNK (**Table 1**). *In vitro*, SP600125 has demonstrated that this inhibitor prevents βAPP induced neuronal cell death as well as down-regulation of ASK1 in F11 cell-lines (Hashimoto et al., 2003). Interestingly, the study did not find neuroprotection against βAPP induced neuroapoptosis when exposing cultures to a p38 inhibitor, which highlights the importance of JNK within this process. Other *in vitro* experiments showed decrease of Aβ-induced cytokine expression (IL6, IL8, MIP1β, TNFα, Groα, GM-CSF; Bamji-Mirza et al., 2014). *In vivo* studies have shown that intracerebroventricular administration of SP600125 improved escape latency in the Morris Water Maze (Ramin et al., 2011). In AD transgenic mouse model (APPxPS1), administration of SP600125 improved spatial learning impairment in the Morris Water Maze, and reduced pTau and Aβ oligomeric burden (Zhou et al., 2015).

### Mixed Linage Kinase Inhibitors

As already mentioned, MLKs have been thought to be a plausible target. Their inhibition could lead to down-regulation of the JNK signaling pathway resulting in antiapopototic and neuroprotective outcomes within neuropathological models. As a result of this, different MLK inhibitors have been developed in order to assess their potential role as a possible therapeutic agent in different pathologies, such as AD (**Table 2**) or Pick’s disease.

The indolocarbazole K252a was the first MLK inhibitor found in *Nocadiopsis* sp. and it has been frequently used in experimental models implicating JNK signaling. In cell culture studies, it has been shown that K252a conferred neuroprotection to Aβ-exposed hippocampal cells (Goodman and Mattson, 1994) and prevented Aβ-induced neuroapoptosis (Xu et al., 2009) which could be of potential benefit in AD. The compound CEP1347 derives from K252a by addition of two ethylthiomethyl groups (Saporito et al., 2002) and acts over MLK1, MLK2, MLK3, DLK (dual leucine zipper kinase) and LZK (Leucine zipper-bearing kinase; Bogoyevitch et al., 2004). CEP1347 reached clinical phase studies (Parkinson Study Group, 2004; Parkinson Study Group PRECEPT Investigators, 2007; Schwid et al., 2010) for the treatment of Parkinson’s disease (PD; Wang et al., 2004). Unfortunately, the results were disappointing. Regarding AD, CEP1347 has been shown to prevent Aβ-induced neuronal cell death and it reduced caspase-3 activity (Bozyczko-Coyne et al., 2001; Troy et al., 2001).

CEP11004 is another carbazole-derived MLK inhibitor that has proved to be useful in PD models, as this compound prevented 6-hydroxydopamine-induced neuroapoptosis in neurons of the *substantia nigra* (Ganguly et al., 2004) and it also appeared as a good inhibitor of the JNK cascade in a MPTP-induced cellular stress model (De Girolamo and Billett, 2006). Further studies will be needed to evaluate the effects of CEP11004 in AD experimental models for its possible relation with the AD-related pathological mechanisms explained above.

URMC099 is a novel MLK inhibitor with good blood-brain-barrier-penetrating properties which has already been shown to be useful in reducing inflammatory response both *in vivo* and *in vitro* models (Goodfellow et al., 2013; Marker et al., 2013). However, no studies have been performed up to date to evaluate the effects of URMC099 in neurodegenerative models.

In summary, although the use of MLK inhibitors has been limited in the AD field, further studies are expected to come.

### Cell-Permeable Peptide Inhibitors

Peptide inhibitors of JNK are peptide sequences that specifically bind to the JNK binding domain (JBD) leading to its inhibition (Bogoyevitch et al., 2004; Borsello and Bonny, 2004). Their characterization came primarily from studies that confirm the interaction of highly expressed JIP1 with JNK, showing that high concentrations of JIP1 are able to induce inhibition of JNK and down-regulation of JNK substrates (Dickens et al., 1997). A conserved 21 aminoacid long sequence was firstly identified within JNK’s primary protein conformation at position 143–163 (Barr et al., 2002; Bogoyevitch et al., 2004). This region is widely known to be the JBD where JIP1 mediates down-modulation over JNK, leading to its inhibition. Purification of the 143–163 region and synthesis of the polypeptide out of this sequence, named I-JIP, showed the capacity of triggering inhibition of JNK. Moreover, a shorter polypeptide obtained from the sequence specified in-between 153 and 163 demonstrated to exert the minimal inhibitory effect on JNK. This compound receives the name of TI-JIP (Barr et al., 2002; Bogoyevitch and Arthur, 2008).

However, the disadvantages that result from the relative non-permeability of JIP need to be solved. Cell-penetrating peptides (CPPs) are small peptides (typically 5–25 amino acids), which are used to facilitate the delivery of normally non-permeable cargos such as other peptides, proteins, nucleic acids, or drugs into cells (Meloni et al., 2014). Hence the observation of Borsello et al. (2003) of attaching permeabilizer-compounds to JIP, such as TAT 48–57 or antannapedia, with the objective of facilitating the diffusion of peptides through membranes in order to exert their action over the desired targets. In this way, different post-modifications were performed that led to the synthesis of the JNK inhibitors JNKi. Furthermore, the *in vitro* synthesis of these compounds using pure D-isomers with the intention of preserving protein functionality and avoiding proteolytic instability, led to obtaining of D-JNKi1 and its L-isomer (L-JNKi1; Borsello and Bonny, 2004). A peptide inhibitor of c-Jun has also been synthesized, the Tat-c-Jun peptide (Holzberg et al., 2003; Antoniou et al., 2011). In this scenario, peptide inhibitors show themselves as promising molecules for targeting JNK, as these compounds have the advantage of specificity toward other kinases (Barr et al., 2002). In fact, one of the most important disadvantages shown by other synthetic JNK inhibitors, such as SP600125 or MLK inhibitor, is their lack of specificity toward their target (Bogoyevitch et al., 2004).

JNK-interacting protein derived compounds have been studied for their possible role in preventing neurodegenerative pathways in which JNK has been shown to be implicated. TAT-TIJIP (Tat cell transporter sequence-bound truncated form of I-JIP) has been demonstrated to be able to prevent neuronal apoptosis via JNK inhibition. TAT-TIJIP effectively prevented cell death by interfering with several processes which have been identified as leading to cell death by necrosis. In particular, reactive oxygen species production was reduced and the increase in cytosolic calcium following the excitotoxic insult was attenuated. These neuroprotective properties of JNK peptide inhibitors likely reflect their abilities to prevent cell death by necrosis as well as apoptosis (Arthur et al., 2007). In different studies the neuroprotective efficacy of four CPPs, namely TAT, penetratin, Arg-9, Pep-1 was shown in a glutamic acid, kainic acid and *in vitro* ischemia injury model (Meloni et al., 2014). AP-1 inhibitory peptides (both full-length and truncated) have also shown neuroprotective efficacy in kainic acid and glutamate neuronal excitotoxicity models (Meade et al., 2010). However, it is to note that TAT-like peptides and other non-related CPPs possess intrinsic neuroprotective properties (Meloni et al., 2014) and pose the question of the contribution of the CPP versus cargo in the neuroprotective effect.

D-JNKi1 is the most frequently used inhibitor in experimental neurodegenerative models. It has been shown useful to reverse ischemia-induced neuronal damage (Borsello et al., 2003). It has been demonstrated that D-JNKi1 is able to decrease levels of APP in human neuroglioma H4 cell lines with the consequent reduction of the βAPP levels and Aβ burdens, and it also shifted APP processing toward the non-amyloidogenic pathways, promoting this non-amyloidogenic processing (Colombo et al., 2009). Again, these events are of high interest as they are directly related to the central pathogenesis of AD.

Regarding AD models (**Table 2**), different studies have confirmed the potential therapeutic benefit of inhibitors for their capacity to interact within a wide variety of molecular signaling processes implicated in this pathology. Sclip et al. (2011) pointed out the efficacy of D-JNKi1 in the mice-based AD model TgCRND8, in which it demonstrated to prevent JNK action leading to rescue memory impairments (behavioral studies) as well as the LTP deficits of TgCRND8 mice. Moreover, D-JNKI1 inhibited APP phosphorylation in Thr-668 and reduced the amyloidogenic cleavage of APP and Aβ oligomers (Sclip et al., 2011). Tran et al. (2012) demonstrated that D-JNKi1 mediated down-regulation of JNK prevented Tau phosphorylation in an AD transgenic model (PS1xAPPxTau). D-JNKi1 has also proved beneficial in another transgenic mice model of AD (TgCRND8), by rescuing synaptic loss and potentiating LTP (Sclip et al., 2014). The increase in pTau levels and neuronal cell death shown in a stress model of AD was also reversed by administration of D-JNKi1 (Solas et al., 2013a). In this scenario, peptide inhibitors could represent a good therapeutic option for the continuously widening therapeutic armamentarium in AD.

### Natural Inhibitors

Three different compounds can be mentioned in this section: latifolians, ω-fatty acids (ω-FAs) and curcumin. Latifolians A and B are natural compounds isolated from the stem bark of the Papua New Guinean vine *Gnetum latifolium* that have been identified as inhibitors of JNK3 (Rochfort et al., 2005). However, no studies have been performed assessing the possible use of latifolians as neuroprotective agents in neurodegenerative models.

On the other hand, the identification of ω-FAs as JNK inhibitors (Ma et al., 2009) could represent a new therapeutic opportunity in AD. The implication of PUFAs (polyunsaturated fatty acids) in neurodegenerative diseases is currently widely accepted. Changes in the lipid-metabolism as a source for reactive oxygen species production and the implication of a dys-homeostasis within the regulation of cholesterol-derivates have been described to play an important role in the development of AD (Corsinovi et al., 2011). In fact, an adequate ω-FAs/cholesterol ratio plays an important role in the regulation of APP-processing pathway, and it has also been suggested that a low consumption of ω-FAs could lead to a major up-regulation of proinflammatory responses (Corsinovi et al., 2011). The administration of ω-FAs prevented IRS-1 (insulin receptor substrate-1) inactivation and pTau pathology by inhibition of the JNK signaling in *in vitro* (neuronal cultures from embryonic Sprague Dawley rats), *in vivo* models (3xTransgenic AD mice) and post-mortem human AD samples (Ma et al., 2009). Altogether it suggests the importance of further studies which could confirm the beneficial outcomes of the use of ω-FAs in the histopathological processing of AD.

Curcumin is a natural compound which resides in the Zingiberaceae sp. family. Aside from its implications as an anti-inflammatory and antioxidant agent, curcumin has also demonstrated to play a direct role in the modulation of the JNK pathway (Chen and Tan, 1998). As a result, different studies have proposed an underlying role of curcumin toward the inhibition of JNK, demonstrating its capacity to ameliorate MPTP (1-methyl-4-phenyl-1,2,3,6-tetrahydropyridine) and MPP^+^ (1-methyl-4-phenylpyridnium) induced neuronal loss models both *in vivo* and *in vitro* (Yu et al., 2010). It also promotes an increase in the expression of HSPs (heat shock proteins) that are centrally implicated in preserving the functionality of the proteasome-mediated degradation of abnormally misfolded proteins (Maiti et al., 2014).

Regarding AD models (**Table 2**), curcumin showed a significant reduction in Aβ40 and Aβ42 levels within the hippocampal structures in APPswe/PS1 mouse after 6-months follow up (Feng et al., 2014) as well as a reduction in Aβ levels and senile plaques histopathology in Tg2576 mice model (Yang et al., 2005). In addition to this, it also demonstrated a significant improvement of the spatial learning and memory ability after a 3-months dosage regimen, as well as a reduced expression of presenilin 2, and an increased activity of Aβ degrading enzymes such as neprilysin (Wang et al., 2014). In fact, combination of docosahexanoic acid (ω-3 FA) and curcumin showed reduced phosphorylation of JNK and tau as well as a decreased degradation of IRS1 in 3xTg AD mice, leading to an Y-maze performance improvement due to a possible role of curcumin in an insulin-sensitization process which directly supports and preserves the insulin tropism within cerebral tissue (Ma et al., 2009). In this way, curcumin could be considered an encouraging proposition as a therapeutic potential drug in AD.

## Conclusion

The JNK cascade is nowadays understood as an axis in the molecular development of AD and other neurodegenerative pathologies. Its implication at different stages of the disease makes clear its importance within neuronal dysregulation, metabolic disruption as well as in formation of pathological structures. Nowadays, different pharmacological agents are available for experimental and preclinical use assessing the possible role of JNK as a plausible therapeutic target in AD. Significant progress in the design of selective JNK inhibitors versus other kinases has been achieved within the past years. However, directed inhibition of JNK isoforms in specific tissues is still an open task. Newer compounds are being developed with increased specificity toward JNK inhibition (Uitdehaag et al., 2012). The fact that JNK3 is specifically expressed in the CNS and its activation by stress-stimuli renders it an attractive and potential target for treating AD. It is possible to speculate that JNK3 specific inhibition will reduce the possible side-effects of a systemic JNK inhibition. Although there is no consensus in literature whether isoform selectivity is needed for the treatment of AD, the answer to this question can only be obtained when such compounds are available.

## Author Contributions

RY performed the bibliographical research and wrote the initial and final draft of the manuscript; SV performed the bibliographical research and wrote the initial and final draft of the manuscript; MS performed the bibliographical research and wrote the initial and final draft of the manuscript; MR organized the manuscript, performed the bibliographical research and wrote the initial and final draft of the manuscript.

## Conflict of Interest Statement

The authors declare that the research was conducted in the absence of any commercial or financial relationships that could be construed as a potential conflict of interest.
